# An Address to the Graduating Class of the Atlanta Medical College

**Published:** 1887-04

**Authors:** Henry Clay Morrison

**Affiliations:** Atlanta, Ga.


THE ATLANTA MEDICAL AND SURGICAL JOURNAL.

VOL. IV. APRIL, 1887. No. 2.

### Original Communications.

### AN ADDRESS

DELIVERED TO THE GRADUATING CLASS OF THE ATLANTA MEDICAL COLLEGE, AT DE GIVE'S OPERA HOUSE, MARCH 4TH, 1887, BY HENRY CLAY MORRISON, D. D., ATLANTA, GA.

While the speculatist inquires for the origin of evil the philanthropist seeks its cure. Whether we came up from the ape is of less moment than whether we are going down toward the ape. The question is not whether a man is an improvement upon his grandfather, but is he a perpetual improvement upon himself. The destruction of evil is the problem of the ages, and the human and divine are confederate in the war. The two natures combine in the God-man to that end to destroy the works of the devil. Life, therefore, is a theatre of antagonisms, and everything has its opposite. Light against darkness, vice against virtue, disease against health and life against death, and each of the laudable professions is a division of the grand army marshaled against evil. The farmer is at war with the thorn and the bramble, the teacher at war with ignorance, the physician with disease, and the minister with sin.

So I address you, young gentlemen of the graduating class, as a body of volunteers just entering the campaign, and with a noble chivalry consecrating life to the rescue of your fellows from the thralldom of disease. Your profession is noble, its responsibilities grave, its importance prime. Entering it, you come into line with names that are imperishable. Even civil liberty has among her champions no names that will outlive those of Hippocrates, Herophilus, St. Luke, Galen, Hunter, Jenner and others down to the Motts and the Dudleys of a later period. Then, like the representatives of the "Lost Cause," should defeat or even failure overtake you, you will have no reason to blush for your leaders.

### THE ENEMY YOU GO TO MEET.

The enemy most to be dreaded is the enemy concealed. Such is disease. Defined as "a deflection from the line of health," it is an ideal, intangible, invisible, and yet a fearfully real thing. In its very subtlety lies much of its danger. Its presence to be detected and cognized only by its work. Hence it must needs be underway with its work of destruction ere its presence can be known. Its most deadly advance like the midnight assassin, with muffled feet and spirit tread. Add to this the fact that it has ten thousand forms, changeful as chameleon hues, and we see that wisdom akin to the divine and insight as the "discernment of spirits" are the need of every Æsculapian knight.

Could disease be brought under law as nature is under law, it were different. Nature is ever true to herself, and in her system law is supreme, like ever producing its like. Hence we sow and reap and ever know what to expect, and knowing what to expect, we invent machinery to meet the expectation, to reap the grain, garner the corn and press the hay.

But disease is a state of unnature—a deflection from nature—not subject to law, neither indeed can be. Disease is the offspring of sin, and sin and its whole brood are insubordinate to law. Sin is the only thing in the universe not under law. Hence we can invent no machinery for culture and cure in the wards of this old world's hospital. Each case is to itself and must have diagnosis and treatment to itself; for while diseases may be classed and named, patients with the same disease cannot be classed. Therefore, young gentlemen, you will have need of perpetual study and investigation. Every new case will demand a new course. Your pastime will be limited indeed. You will have little need of fancy fishing tackle, three hundred dollar shotguns and five hundred dollar setters. The only doctor with time for idleness and pleasure is the doctor without patients, and the doctor who is much in pastime and idleness will be perpetually patientless. Your profession places you at the service of the suffering, and they will demand your time in proportion to what you are worth to them.

### THE MUNITIONS OF YOUR WARFARE ARE GOD-PREPARED.

Nature, as if conscious of her children's need, is ever running her mighty laboratories, preparing the elements with which disease is to be combatted. The mineral and vegetable kingdoms kneel at the feet of your profession. Materia medica, with untold supplies, stands at your elbow, while the chemist is co-worker between nature and the physician. Nature, in her secret chemistries, produces the elements, the practical chemist refines and prepares them and the physician, skilled in their use, employs them for the reduction of disease. Thus the partnership is close and the dependence mutual. Every man in the practice of medicine, whether he be saint or scientist, is a co-worker with nature and with God for the destruction of the common enemy, disease; and God has as verily a part in every drug-store, and as vital connection with every medical office as he has with the family altar or the pulpit; and I rejoice that the day has come when professional men realize and recognize their high position as a part of the one great army arrayed against sin and its consequences.

Your term in this college has been your *drill period.* Guided by wise and competent preceptors, you have gone through the preparation and completed the drill—finished the task of physiological, anatomical and chemical investigation. You are now supposed to know every bone and muscle, nerve and tendon of him who is so "fearfully and wonderfully made." You have also theoretic knowledge of the army of diseases that seek his destruction. You know somewhat of their mode of attack and the munitions needed to repulse them. You have gone through the whole curriculum and have to-night received parchments certifying to the same.

Yet allow me to say, you are not yet physicians. A volunteer is one thing, a veteran quite another, and many a volunteer never becomes a veteran. You are not yet in sight of Troy; you have met no Trojan yet; you have received no spear-thrust upon your sheep-skin shields, and while that diploma (well-won) will grace your wall and bear proof of your faithful college-work, it will parry no enemy's dart. "D. D." has ruined many a preacher, but never made one; and while I will not take liberties with a profession to which I do not belong, I will say that though "M. D." may never have spoiled a doctor, I am sure it never made one.

You go now from your *alma mater* to the field. Drilled, armed, equipped, diplomaed, saddle-bagged, mounted, you advance upon a future more dependent after all upon "the stuff you are made of," the elements of your native, inborn selfhood, than upon the honors which crown you at this hour. Still I do not discount your present honors, but congratulate you, trusting that the success of the hour may be the inspiration and the prophecy of succeeding successes that shall mark that future to which you go; and I bid you go in that spirit of resolute determination before which enemies quail and obstacles cannot stand. Go to succeed. Let your motto be:

> " Better, like Hector, in the field to die,
> Than, like a perfumed Paris, turn and fly."

### THE ELEMENTS OF SUCCESS.

Voltaire says " nothing is more estimable than a physician who, having studied nature from his youth, knows the properties of the human body, the diseases which assail it, the remedies which benefit it, exercises his art with caution, and pays equal attention to the rich and the poor." The Hippocratic system taught, that the "four humors," rightly proportioned and diffused through the system, formed the foundation of health, and so there are four things needed in right proportion for success in your profession: *Knowledge*, *caution*, *courage* and *self-sacrifice*. These are as essential to your success as the "four humors" to the health of your patients.

The issues at stake demand thorough knowledge. Life, with all it implies, is placed in your hands. Ignorance, therefore, can be no less than crime. Accepting the responsibility of the case without the requisite knowledge, and death resulting from that want of knowledge, your crime can be no less than homicide; and whether you have the needed knowledge the sufferer cannot judge, but in his suffering confides all to you; and though men may criticise you and have sport at the expense of your profession, yet they will call for you in the solicitous hour. And we must consent to be laughed at. It makes the world better to laugh; hence, the man who makes the world laugh is a benefactor. The facetious poet produced a smile at the cost of your profession when he advocated the propriety of a partnership in the practice, saying :

> "See one physician, like a sculler plies,
> The patient lingers and by inches dies;
> But two physicians, like a pair of oars,
> Waft him more swiftly to the Stygian shores."

But in the hour of suffering and distress the spirit of levity departs, and you will be called for and life placed in your hands, and fondest hopes hang upon your skill.

> "Physicians mend or end us, *secundem artem*,
> But though we sneer in health,
> When ill, we call them to attend us,
> Without the least propensity to jeer."

And the man, without proper knowledge, grasping the vessel's helm to steer 'twixt Scylla and Charybdis, is a madman. The man ignorant of an engine, and putting his hand upon the throttle to run a train through midnight dangers, is a moral desperado; but the man who enters the home and takes the life of the husband and father in his hands, while wife and little ones look to him as little less than a Saviour, and is unfurnished with the requisite knowledge for the emergency—such a man is a fiend that hell itself might blush to recognize.

The lawyer, doctor, preacher—too indolent for perpetual study! A beautiful trio, indeed. *Lexico*, *medico*, *clerico* pirates, clad in the livery of pretended learning, with canvas of high-sounding titles, sailing the high seas of the professions.

Caution and courage will also be in constant requisition. Few men, like Goethe, ascend to the first places at a single bound and retain their position undisputed. But when you have attained to success you will find your path has been as that from Chattanooga to the Gate City—every foot of it fought over. Caution is ever essential where dangers are so many and so subtle. The unfortunate Braddock met his memorable defeat, not because triumph was impossible, but for want of caution and disdaining advice. An engineer misread his order by one single letter, dashed forward at a fearful speed, and in eight minutes two locomotives were in collision, meeting with such force as to leap (as two huge beasts in conflict) twenty-five feet into the air, falling back a mass of debris. The soul of that engineer, together with three others, were sent into eternity, while your speaker himself was in the wreck. There are tragic moments before you in your profession—moments when you will stand with finger upon the very springs of life—the soul of your patient, like a captive bird, in your grasp. Caution, then, like an invisible and divine hand, may turn disaster aside and bring to you the glory of success.

You will also need that *courage* that *braves defeat.* There will be hours when this must be your support. Failure, defeat, disaster have made part of the experience of the grandest men of the ages, and we learn through failure how to conquer success. Demosthenes was hissed from the presence of the multitude. D'Israeli retired chagrined and humiliated from before the parliament. Generals Grant and Sherman came near being removed from command for their mistakes, and Grant had been consigned to disgrace but for the heroic efforts of his friend, General Washburne. Bishop Simpson and others of the first orators of the church overcame defects and triumphed over obstacles, making of them stepping-stones on which to ascend to undying fame. Then, young gentlemen, in the hours of failure and misfortune (the test hours, the hours that determine the *quantum* of manhood), when the black vulture of adversity has flopped you down and seems as if he would beat out your very powers with his merciless wings, remember at such a juncture that you are in the pathway trod by the noblest sons of success, and that you are but fighting the same fiends they have fought and overcome.

### SELF-SACRIFICE.

This divine principle underlies all redemptive economy, and must be a prime element in every grand life—to rise above self and selfish motives and live and labor and suffer for others. I care not what may be your notions of theology or your professional qualifications, your life-work will be a success and your name will live in proportion to the self-sacrifice you put into life. To use, in part, the thought of another: A man may begin life as the worm begins it, and feed voraciously until full; then slough his worm-skin and spin round him a silken house and thus retire from life. This will do for a bug; but that which is well for a bug is a poor thing for a man. The chrysalis is not a fool, for there is a next summer for him; but the man will never hatch nor be so much as a butterfly, but he rots in his self-spun cocoon, and his name rots with him.

He who said, "*Talitha cumi*," and the damsel arose—the touch of whose garment lifted up the twelve-years-bowed-down—He built his life-work on this principle of self-sacrifice; and they who mockingly said, "He saved others, himself he cannot save," uttered a profound truth, the magnitude and depth of which they knew not. Of course He could not save others and himself also, but He saved others by giving himself; and it is as true of you, young gentlemen, as it was of the Nazarene. You cannot save others but by giving yourself.

ALL TRUE LIFE IS VICARIOUS.

The miner in the shaft, with lamp and pick, is giving his life for others; the sailor, who accepts the waves as his home; the policeman, treading his midnight beat, guarding your city; the man in public trust, who is faithful to his duty—all are giving life for others and for the public good; and as you go to the practice of your profession and become a part of the great, grinding, battling world, you go in the interest of others.

Your support and remuneration, though a matter of moment, is not to be the first consideration. "The laborer is worthy of his hire." It is the divine design that your endeavor shall not go unrequited; but there is a chivalry prompting every true man higher than the mere chivalry of dollars and cents, and you are to go into your life-work to bless others – to relieve suffering, and soothe sorrow, and lift up humanity to a higher and happier plane; and you will do this through foregone pleasures, broken rest, over-taxed powers, and amid noonday heat and midnight storm. At such self-cost as this you are to carry relief to the sufferer and the light of hope to loved ones in despair. Castilian knight, clad in armor of steel and breasting the night-storm to reach and rescue the helpless, was not grander than the true physician, pressing his way through the warring elements of the starless night to reach the family in distress; and you will do your work by a perpetual self-investment, giving self to save others. History immortalizes the names of those who die on their country's altar; but your profession has produced its heroes as true, and I doubt not there will come from your own class as true heroes as ever fell on hard-fought field.

THE NATURE OF YOUR WORK.

It can but be momentous since it has to do with life—to prolong life and make it what life should be. Disease introverts the thought. It turns a man's thoughts in upon himself, and self-thought tends to selfishness, narrowness and moral dwarfhood. It is not in man to lose sight of self when suffering; and the man who relieves the sufferer turns him from self-thought and makes him a nobler, broader and better man. Hence your work, as physicians, goes further than flesh, and bone, and blood, and muscle. It is not an absolute materialism, but it takes hold on the higher nature and the higher interests of those whom you heal. You will change sighs into songs, tears into smiles, despair into hope, and the shades of solicitude into the sunshine of assurance.

It is a *remedy* that our race needs. "The whole creation groaneth and travaileth together in pain," and the man is a benefactor who makes its suffering less. Humanity, in its anguish, will greet and bless you as you bring it a remedy. The man whom you raised from what seemed the bed of death will give you first place in his heart, and his wife and children, saved from widowhood and orphanage, will speak your name tenderly through coming years. Heal a man, "ease his pain, and you have an influence over him for all time." Every true physician is a prince. He has the hearts of those whom he benefits. No men have the confidence of their patrons so full and complete; hence none have such influence as you will wield. I address a company of princes prospective, destined to rule, in the deepest, tenderest and most real sense, the people among whom you live and labor.

Finally, young gentlemen, allow me to say (and I ask no apology for the utterance), you of all men, excepting the clergy, should have the *Christ-life.* You should know the things of that higher sphere, whose life and laws are not the themes of your professional text-books. There is a limit to your skill and a limit to human life, and destiny and relentless time drive doctor and patient ever toward that limit. Over and above your best skill and effort stands the immutable "appointment unto man once to die;" and in the silent, solemn moment when that limit is reached, and you resign your patient to the inevitable—when life ebbs and hope dies, and tears fall, and hearts bleed—then to be a "comforter" in such an hour is a grander and a diviner ministry than materia medica can supply. The physician who, when he has done all that skill can do, can then lay aside his medicine-case, and, taking the old family Bible, with its blessed light and solid promises, can lead the disconsolate through the shadows to comfort, resignation and rest, he it is who has most of that manliness that characterized the only perfect Man; and now the heart-wish of your speaker is that success may be yours from this hour of honor until you reach the time-limit of which we have spoken; and when that limit is passed,

> "Then may you join the choir invisible
> Of those immortal dead who live again
> In lives made better by their presence; live
> In pulses stirred to generosity,
> In deeds of daring rectitude, in scorn
> For miserable aims that end in self,
> In thoughts sublime that pierce the night like stars,
> And with their mild persistence urge man's search
> To vaster issues."

